# Steel work design, production and analysis of a fish feed mixing machine

**DOI:** 10.1016/j.heliyon.2021.e07658

**Published:** 2021-07-24

**Authors:** Paul Chukwulozie Okolie, Echezona Nnaemeka Obika, Benjamin Segun Oluwadare, Onyemazuwa Andrew Azaka, Uchenna Onyebuchi Okolie

**Affiliations:** a Department of Mechanical Engineering, Nnamdi Azikiwe University PMB 5025, Awka, Anambra State, Nigeria; b Department of Mechanical Engineering, Ekiti State University PMB 5363, Ado Ekiti, Ekiti State, Nigeria; c Electronics Development Institute (ELDI), KM 80 Enugu-Onitsha Expressway, Awka Capital Territory, PMB 5099, Awka, Anambra State, Nigeria

**Keywords:** Mixer, Feed, Farming, Homogenous, Machine

## Abstract

As Nigeria props up her desire to boost local manufacturing and production industries, the need for proper design of an efficient and reliable fish feed mixing machine for safe and economic use cannot be overemphasized, as an efficient and reliable machine would minimize deterioration and losses during production to deliver good quality products (fish feeds) obtainable at the lowest production cost as well as the shortest production time. A blade-type fish feed mixer has been designed and fabricated. The mixer runs with a 1.5Hp, single-phase electric motor and has a production capacity of 50 kg/h. It was designed with the aid of auto cad software and fabricated using stainless steel and galvanized steel materials. All the fixed metal parts were joined using electric arc welding, while the detachable parts were fastened with an adequate size of bolt and nut. he effect of mixing time and speed on the homogeneity of the feed mix obtained was ascertained using the Central Composite Design tool of the Design-Expert software was used. The result showed that mixing time has a positive effect on the homogeneity of fish feed with its peak at about 25 minutes. the mixing speed also gives a positive effect with its peak at 220rpm. However, mixing speed beyond 220rpm gives a negative effect on the homogeneity of the feed. With the aid of the Auto cad inventor software, stress analyses were conducted on the conveyor assembly, considering gravity forces, body forces, remote forces, and moment. On testing, the efficiency of the machine was evaluated to be 75 %. The finite element analysis gave a maximum von mixes stress of 53.7697 MPa for the assembly which is not up to half of the yield strength of the material used for the machine. Hence, the machine is safe and durable.

## 1 Introduction

Feed mixers are employed in feed mills for the mixing of feed ingredients (Chime et al., 2015). In the production process of fish feed, the importance of the feed mixer cannot be overemphasized, as a good fish feed is the product of an efficient mixer. One of the most important characteristics of a good fish feed is the proper distribution of the feed ingredients. In the traditional setting, crude and primitive mixing techniques employed the use of the hand for mixing of poultry feed, before the introduction of manually operated mixers birthed by the industrial evolution of Great Britain (Onyegu et al., 2012). The most prominent economic activity in rural areas here in Nigeria is agriculture, with livestock farming being a prominent aspect. Presently, the economic situation of Nigeria has seen a massive shift in the interest of citizens in agriculture. This shift in interest has led to the birth of many agricultural-based businesses in urban areas. Fish and poultry farms have increased tremendously in these areas. In the bid to reduce the cost of feed production, farmers become directly involved in the production of the feeds. The production of these feeds involves grinding of the individual ingredients and mixing them properly to get a homogenous mixture (Adenigba and Olalusi, 2019). The importance of achieving a homogenous mix is to meet the nutritional requirement of the fish being raised (Balami et al., 2013). Through the process of extrusion, this mixture is further compressed into pellets for fish farming (Mc-Donald, 1995), as seen in the study of Okolie et al. (2019).

However, the use of primitive mixing techniques is still prevalent among farmers. Due to the low mechanical advantage associated with manual hand mixing, research has shown that its adoption has led to both insufficient fish feed production as well as improper mixing of the produced feed. Thus, for increased rate of feed production as well as the eradication of stresses involved in farm activities, the production of small scale feed processing equipment is paramount. To overcome the challenge of the high cost associated with the production of homogenous feed, small-scale mixers have to be locally produced. Therefore, this study aims to design and construct a motorized blade type mixer, using locally sourced materials to take care of some stress and high cost of production in our farms. To achieve this, proper machine design was carried out to improve the structural integrity of the machine and consequently tackle the issue of failure associated with the existing designs. This will assist, quality assurance engineers, reliability engineers, design engineers, etc. in the development of quality and efficient machine parts (Andrew et al., 2020).

## 2 Materials and methods

### 2.1 Machine parts

To ensure an efficient and homogenous mixture of feed ingredients, a fish feed mixer is employed. The main parts of the fish feed mixer include: The mixing chamber, the blade type conveyor, the frame, the electric motor, and the conveyor assembly shaft.

## 3 Machine design and construction

Solid works software, Autodesk Inventor, and Arc/Gas welding process was employed in the design/construction and analyses of the machine. Solid works software was used for the engineering design, Autodesk Inventor was used in the static and dynamic stress analysis on the conveyor assembly, while Arc and Gas welding process was used in the construction of the fish feed mixing machine. Relevant equations and parameters were also employed.

### 3.1 The drive

Due to its flexibility, simplicity, low maintenance requirement as well as good damping characteristics, the V-belt pulley arrangements system was employed in the machine design, for power transmission from the electric motor to the mixing unit.

### 3.2 Selection of ball bearing

The rolling ball bearing of type 70000AC (46304) which is fitted on both ends of the conveyor assembly shaft was technically selected to avoid failure of the fish feed during operation. Bearing radial and axial loads, including speed of rotation and the required static safety factor were considered. ANSI/AFBMA 9-1990 (ISO 281-1990) calculation method and lubricant (grease) frictional factor μ of 0.0015ul was used in the bearing life calculation under a working temperature of 100 °C. The Loads acting on the bearing, bearing parameters, and bearing life calculation are reported in Tables 1 and 2.

**Table 1:** Loads acting on the bearing.

Parameter	Symbol	Value
Radial load	F_r_	20N
Axial load	F_a_	50N
Speed	n	1500rpm
Static safety factor	S_o_	2.0ul

**Table 2:** Bearing parameters.

Parameter	Symbol	Value
Inner diameter	d	35.000 mm
Outer diameter	D	80.000 mm
Width	B	21.000 mm
Nominal contact angle	α	25 deg
Dynamic load	C	26000 N
Static load	C_0_	17600 N
Dynamic Load Factor (radial)	X	0.60 ul/0.60 ul
Dynamic Load Factor (axial)	Y	0.50 ul/0.50 ul
Static radial load Factor (radial)	X_0_	0.60 ul
Static axial load Factor (axial)	Y_0_	0.50 ul

### 3.3 Pulley diameters

The diameter of the mixing auger pulley is calculated using Eq. (1)

(1) $$D_{2}=N_{1}D_{1}/N_{2}$$

Where: N_1_, N_2_ = speeds of motor and mixing auger respectively, rpm; D_1_, D_2_ = diameters of motor pulley and mixing auger pulley respectively.

$$D_{2}=\;1400\;x\;350\;/\;580$$

$$D_{2}=120mm$$

### 3.4 Belt speed

Shigley and Mischike (2001) gave an expression for the mixer drive belt speed, as seen in Eq. (2).

(2) $$V\;=\;N_{1}D_{1}\;/\;60$$

Where; V = belt speed

D_1_ = diameter of driver pulley

N_1_ = speed of driver in rpm

V=0.05x1400/60

V=1.17m/s

### 3.5 Determination of belt length

With the pulley diameters D_1_ = 350mm, D_2_ = 120mm and motor pulley to shaft pulley distance, C = 460mm, Eq. (3) gives the expression for required belt length (Fenner, 1994).

(3) $$L\;=\;2C\;+\;\left(D_{2}-D_{1}/4C\right)\;+\;1.57\left(D_{2}\;+\;D_{1}\right)$$

Where, C = centre to centre distance between the driver and the driven (460mm).

D_1_ and D_2_ = diameters of the driver and driven pulleys

L=1281mm

The results of theoretical analysis and computation in the Inventor V-belt design generator indicate that the design meets the specified criteria. A narrow V-belt DIN 2215 is designed and selected for this purpose, the belt dimensions are 10mm, 6.0mm, and 8.5mm for width, height, and datum respectively. Belt tension is computed for Pulley 1. Belt strength check calculation indicates design compliance.

### 3.6 Capacity of the conveyor

For the actual mixing, the horizontal blade conveyor in a closed cylindrical barrel system was adopted for the machine design. The auger is pyramidal in shape, consisting of uniform curved edges of 105mm diameter and 80mm pitch. Eq. (4) gives an expression for the auger capacity.

(4) Q=60nΦp(D−d)π/4

Where; Q = conveyor capacity, t/h;

n = number of screw rotations, 580rpm;

p = conveyor pitch, 0.1 m;

D = conveyor pitch diameter, 105mm;

d = shaft diameter, 35mm

Փ = factor of safety for inclined conveyor, 0.33 (Delucia and Assennato, 1994).

Q=63.14t/h

### 3.7 Power required by the conveyor

The expression for required conveyor power is given in Eq. (5)

(5) $$Pr\;=\;QL\left(\omega_{o}+sin\beta {}^{\circ}\right)\;/\;367\;$$

For slow–flowing abrasive material, ω_o_ is 4.0, with 0° conveyor inclination angle (β) and 0.81 conveyor length (L)

Pr=0.44KW

### 3.8 Conveyor assembly stress analysis

**Figure 1:**
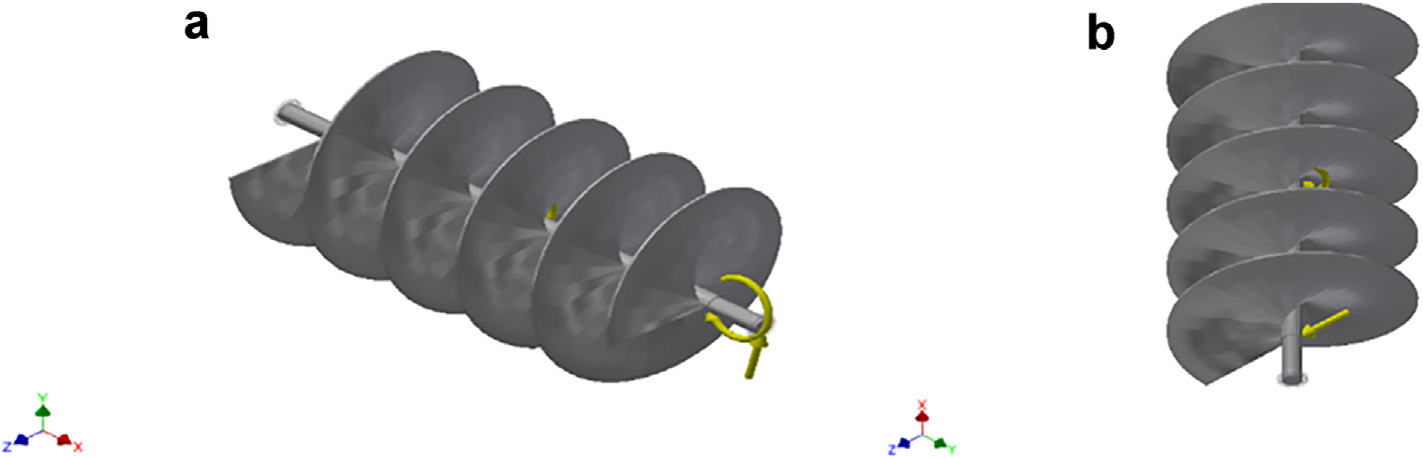
a & b: Gravity force of magnitude 9810 mm/s^2^, body load acting on the selected faces of the conveyor assembly (simulations 1,2,3).

**Figure 2:**
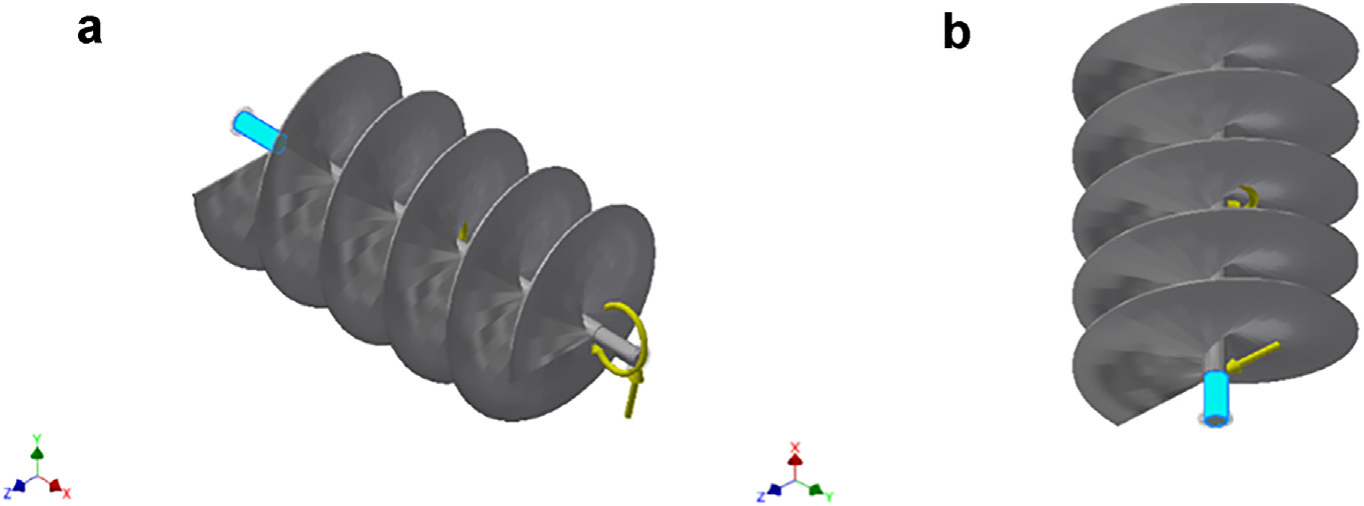
a & b: Remote Force 1 acting on the selected faces of the conveyor assembly.

**Figure 3:**
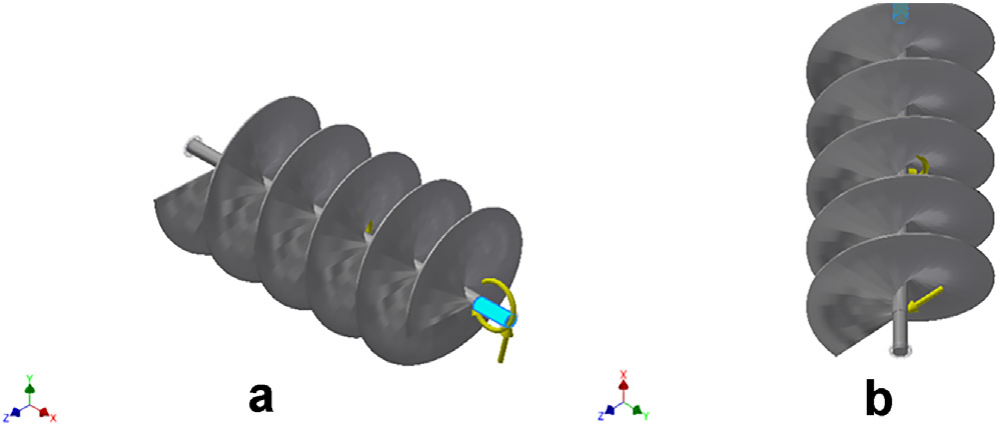
a & b: Remote Force 2 acting on the selected faces of the conveyor assembly.

**Figure 4:**
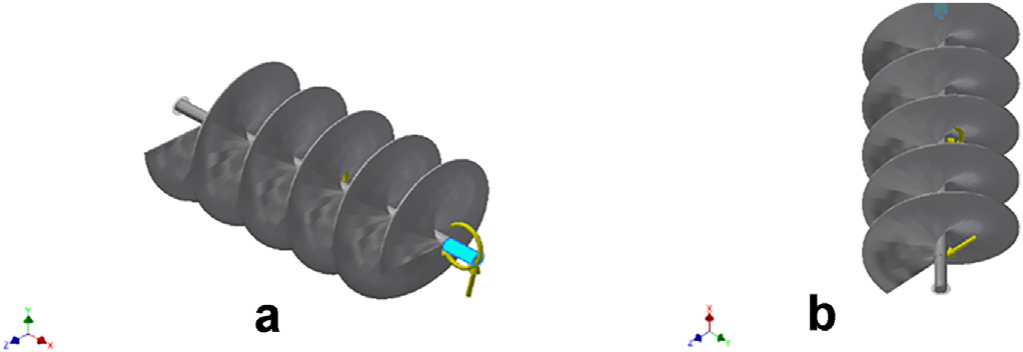
a & b: Reaction moment acting on the selected faces of the conveyor assembly.

**Figure 5:**
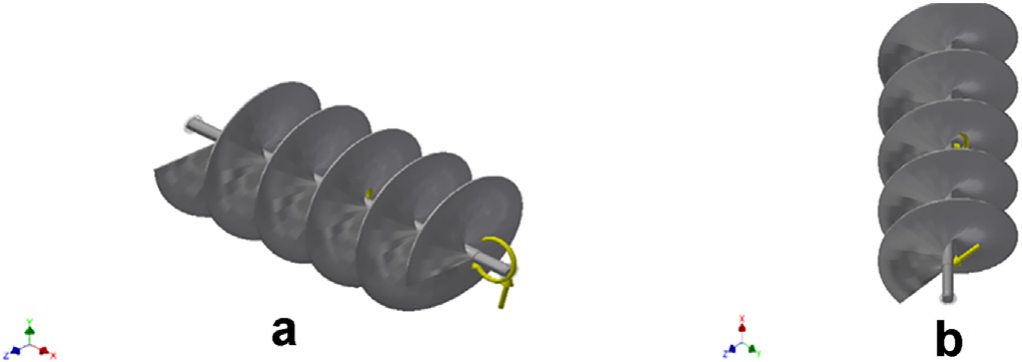
a & b: Body loads acting on the selected faces of the conveyor assembly.

Static and dynamic stress analyses were performed on the fish feed conveyor assembly consisting of the shaft and pyramidal shape blades. The stress analysis was conducted using the AutoCAD inventor. The design objective is a single point static analysis which is to detect and eliminate rigid body modes while considering motion load analysis on the conveyor assembly. Three (3) simulations with different time steps were carried out on the conveyor assembly. This is to ascertain the accuracy of the results. Separate stresses across contact surfaces were not considered in the analysis. However, the mesh settings for the simulations were the same. Operating conditions: The analysis was done on different operating conditions (load types) that could fail the conveyor assembly. The load types considered were: Gravity forces, body forces, remote forces 1 & 2, and moment, as shown in Figures 1 and 2, 3, 4, and 5 respectively. A summary of the remote forces and reactions is given in Table 3, while that of the body loads is captured in Table 4. These are the forces that could cause the assembly to fail. These forces were made to act on selected faces of the conveyor assembly.

**Table 3:** Operating conditions showing gravity, remote forces and reaction moment for dynamic simulation of the conveyor assembly.

Load Type	Gravity	Remote Force 1	Remote Force 2	Reaction Moment
**Simulation 1**				
Magnitude	9810 mm s^−2^	101.949 N	458.446 N	254683.39 N mm
Vector X	0 mm s^−2^	-58.860 N	58.860 N	0 N mm
Vector Y	-9810 mm s^−2^	-56.716 N	450.551 N	-54835.662 N mm
Vector Z	0 mm s^−2^	60.928 N	-60.928 N	-248710.03 N mm
Remote Point X	-	100.6 mm	1000.6 mm	-
Remote Point Y	-	0mm	0 mm	-
Remote Point Z	-	0 mm	0 mm	-
**Simulation 2**				
Magnitude	9810.000 mm s^−2^	101.949 N	458.506 N	254731.985 N mm
Vector X	0.000 mm s^−2^	-58.860 N	58.860 N	0 N mm
Vector Y	-9810.000 mm s^−2^	-56.786 N	450.620 N	-54777.295 N mm
Vector Z	0.000 mm s^−2^	60.863 N	-60.863 N	-248772.651N mm
Remote Point X	--	100.6 mm	1000.6 mm	--
Remote Point Y	--	0mm	0mm	--
Remote Point Z	--	0.mm	0mm	--
**Simulation 3**				
Magnitude	9810.000 mm s^−2^	101.949 N	458.515 N	254739.456 N mm
Vector X	0.000 mm s^−2^	-58.860 N	58.860 N	0.000 N mm
Vector Y	-9810.000 mm s^−2^	-56.797 N	450.631 N	-54768.309 N mm
Vector Z	0.000 mm s^−2^	60.853 N	-60.853 N	-248782.280 N mm
Remote Point X	-	100 mm	1000.6 mm	-
Remote Point Y	-	0 mm	0 mm	-
Remote Point Z	-	0 mm	0mm	--

**Table 4:** Body Loads acting on the conveyor assembly.

Load Type	Body Loads (Simulation 1)	Body Loads (Simulation 2)	Body Loads (Simulation 3)
**Angular Velocity**			
Magnitude	0.026^o^ s^−1^	0.122^o^ s^−1^	0.130^o^ s^−1^
Vector X	-0.026^o^ s^−1^	-0.122^o^ s^−1^	-0.130^o^ s^−1^
Vector Y	0^o^ s^−1^	0^o^ s^−1^	0^o^ s^−1^
Vector Z	0^o^ s^−1^	0^o^ s^−1^	0^o^ s^−1^
Axis Location X	500.6 mm	500.6 mm	500.6 mm
Axis Location Y	0 mm	0 mm	0 mm
Axis Location Z	0 mm	0 mm	0 mm
**Angular Acceleration**			
Magnitude	0.110^o^ s^−2^	0.106^o^ s^−2^	0.105^o^ s^−2^
Vector X	-0.110^o^ s^−2^	-0.106^o^ s^−2^	-0.105^o^ s^−2^
Vector Y	0^o^ s^−2^	0^o^ s^−2^	0^o^ s^−2^
Vector Z	0^o^ s^−2^	0^o^ s^−2^	0^o^ s^−2^

### 3.9 Homogeneity test

The experimental design to ascertain the effect of mixing time and speed (independent variables) on the homogeneity (dependent variable) of the mixture was done using the design expert software. A total of thirteen experimental runs were obtained. To determine the homogeneity (degree of mixture), for the experimental runs, three fish feed ingredients (corn, groundnut cake, and born meal), were used. The corn, groundnut cake, and born meal were ground into 250, 450, and 650μm particle sizes respectively. 4kg of each ingredient were charged into the mixer and three samples (2Kg each) were collected from each experimental run after mixing. The homogeneity of the feed mix was determined using Eqs. 6, 7, and 8, adopted for the study of Adenigba and Olalusi (2019).

(6) $$s=\sqrt{\frac{\sum {\left(X-\bar{x}\right)}^{2}}{\left(n-1\right)}}$$

(7) $$CV=\frac{s}{\bar{x}}$$

(8) H=(1−CV)×100

“CV” is the coefficient of variability, “H” represents the homogeneity expressed in percentage. The standard deviation is expressed as “S” which is the mean weight of groundnut cake from the three samples of each experimental run. “X” is the weight of corn in each sample and “n” is the number of samples collected.

## 4 The fish feed mixer construction

For proper material selection, several factors were put into consideration. The considered factors cut across their strength, machinability, rigidity, availability, as well as cost (Okolie et al., 2015, 2019, 2020). The materials as well as their specifications, utilized for the construction of the parts are listed in Table 5.

**Table 5:** Materials for the fish feed mixer.

Component	Specification	Material	Number of parts
Barrel	300 mm × 800 mm	Galvanized steel	1
Bearing	6015	**-**	**2**
Shaft	35 mm × 125 mm	Stainless steel	1
Frame	120 mm × 570 mm x 525 mm	Angel iron	1
Pulley	35 mm × 12 mm (internal and external diameters)	Cast iron	1
Belt drive	12mm thickness	Rubber	1

### 4.1 Barrel design and construction

An 8mm thick galvanized steel pipe of 300mm diameter was machined to a length of 800mm. For easy maintenance, the barrel was designed to be fastened with bolt and nuts by attaching circular wedges to it on both sides.

### 4.2 Blade type conveyor and shaft construction

**Figure 6:**
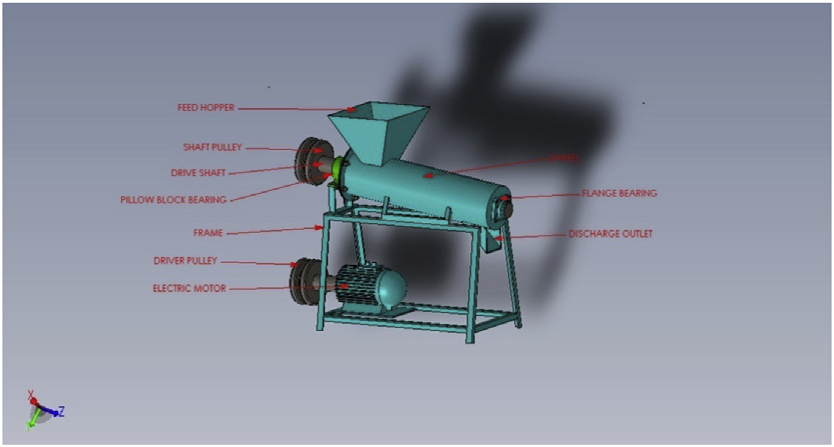
3D model of the fish feed mixer.

**Figure 7:**
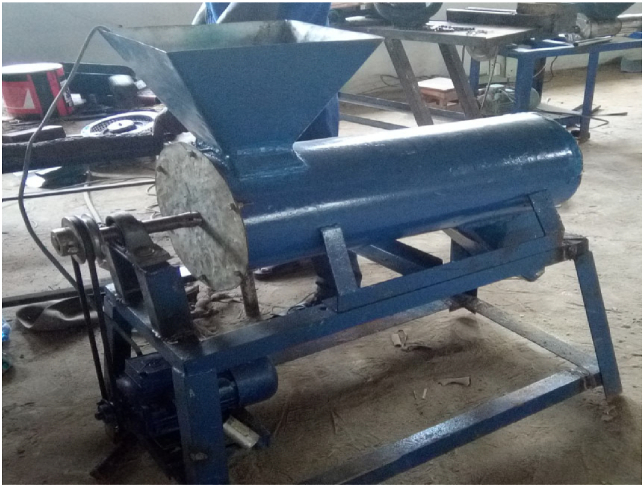
Fabricated fish feed mixer.

It is the combination of pyramidal-shaped blades and the shaft. A 280mm blade type conveyor was constructed by machining the blades to a shaft at a tilt angle of 60°, to obtain a displacement of 10mm between the blades and the barrel. A solid model of the design is shown in Figure 6 while the constructed machine is shown in Figure 7.

## 5 Results and discussion

### 5.1 Results of bearing basic life rating

The load on the bearing resulting from the conveyor assembly shaft and the bearing parameters used for bearing life calculations were tabulated in Tables 1 and 2 respectively. With the bearings nominal contact angle of 25 °C, dynamic load (C) rated 26000 N, static load (C_O_) rated 17600 N, life rating ($L_{req}$) at 100000hrs, required reliability ($R_{req}$) of 90ul, a factor of 1 for both life adjustment of bearing properties as well as that of operating conditions, 100 °C working temperature, and factor of additional forces f_d_ of 1. The strength check was seen to be positive when the factors for the radial load (X_O_) and axial load (Y), for static and dynamic conditions respectively were 0.6ul and 0.5ul.

### 5.2 Results of the reaction force and moment on constraints stress analysis for the conveyor assembly

**Figure 8:**
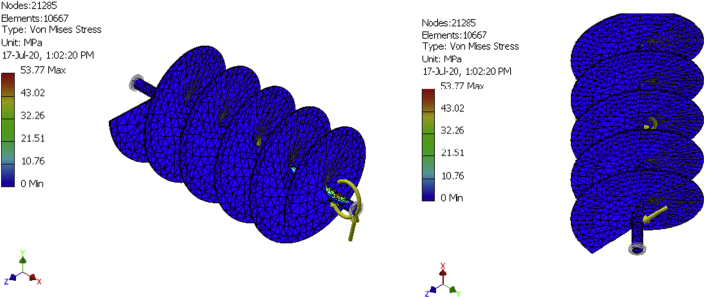
FEA simulation result of the Von Mises Stress on the selected faces of the conveyor assembly for simulation 1.

**Figure 9:**
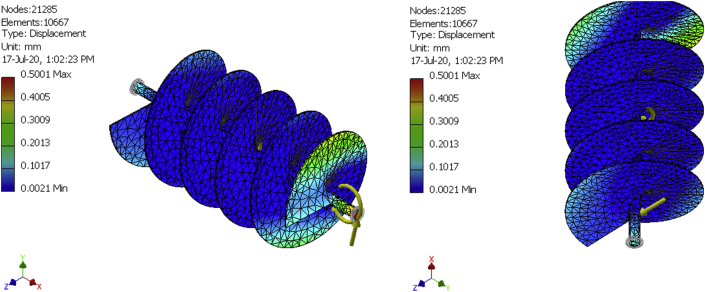
FEA simulation result of the displacement on the selected faces of the conveyor assembly for simulation 1.

**Figure 10:**
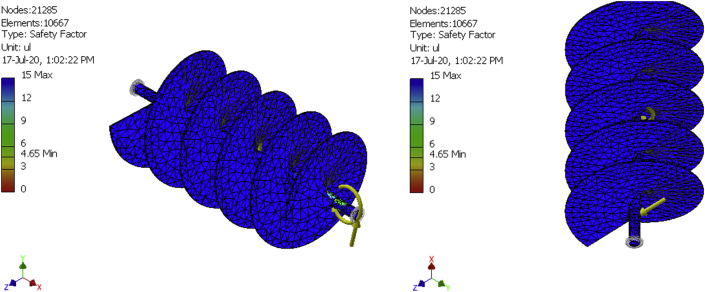
FEA simulation result of the safety factor on the selected faces of the conveyor assembly for simulation 1.

**Figure 11:**
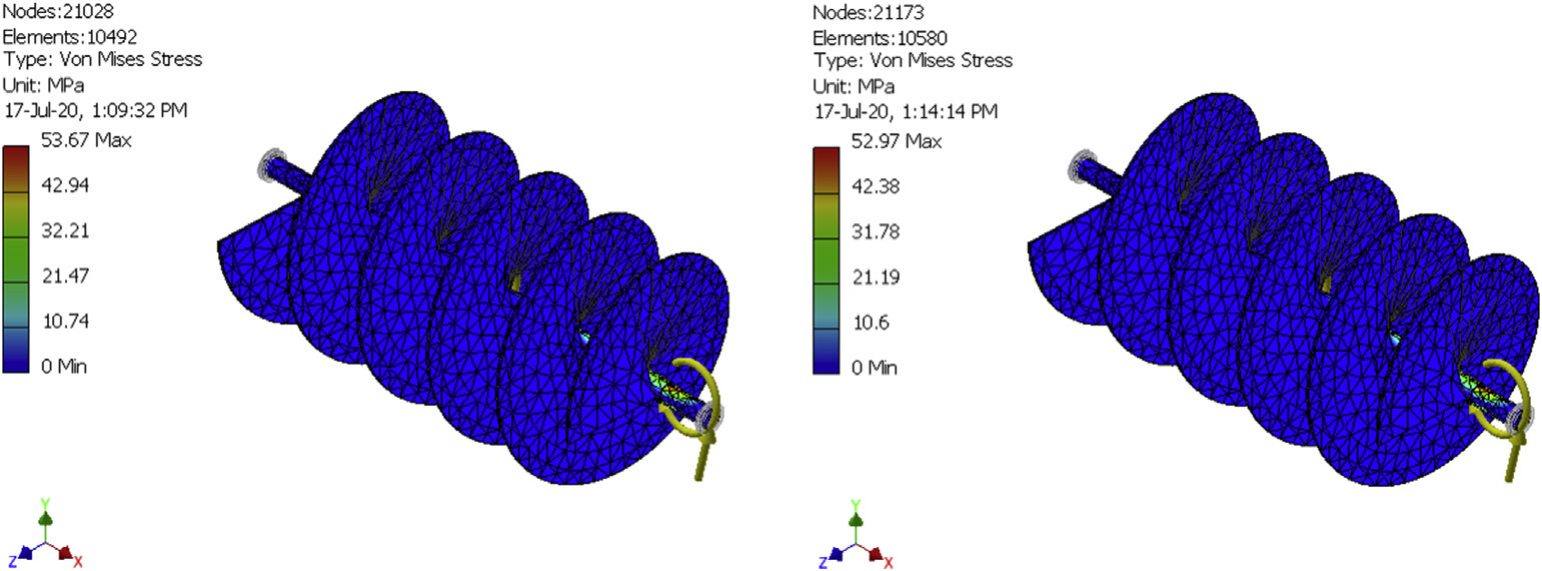
FEA simulation result of the Von Mises Stress on the selected faces of the conveyor assembly for simulations 2 and 3.

**Figure 12:**
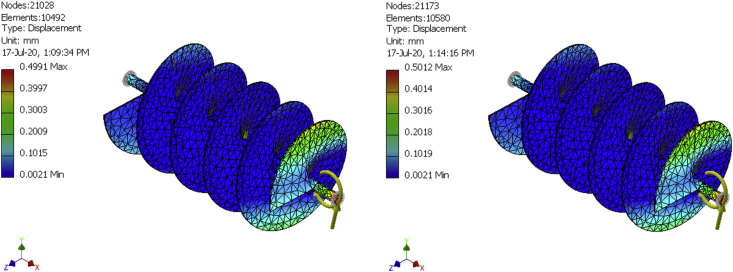
FEA simulation result of the displacement on the selected faces of the conveyor assembly for simulations 2 and 3.

**Figure 13:**
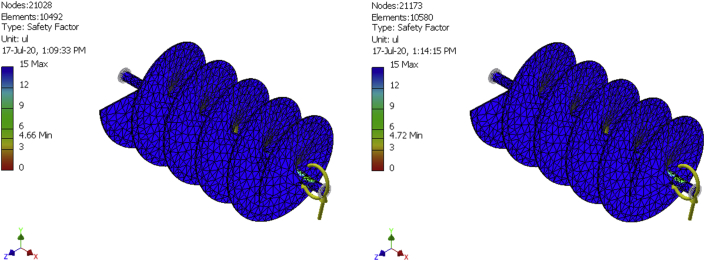
FEA simulation result of the safety factor on the selected faces of the conveyor assembly for simulations 2 and 3.

In finite elements analysis, solutions can be improved by mesh refinement. That is why meshes were generated for a 10,492-element model, a 10580-element model, and a 10667-element model as shown in Figures 8, 9, 10, 11, 12, and 13 for the three FE simulations to show that the solutions progressively fell within the design parameters as the number of elements improved. The forces, 101.949N and 458.446 N were used to run a simulation study on the conveyor assembly and the obtained result is shown in Table 6. The von mises failure criteria was used to examine the strength of the assembly, from the study, the maximum von mises stress value is 53.7697 MPa. Since the von mises stress value is not up to half of the yield strength of the material and the factor of safety is 15, the design of the component is considered safe.

**Table 6:** Simulation result summary of the conveyor assembly.

Parameter	Minimum	Maximum
Volume	4986680 mm^3^	
Mass	40.1429 kg	
Von Mises Stress	0.00174919 MPa	53.7697 MPa
Displacement	0.0020779 mm	0.500052 mm
Safety Factor	4.64946 ul	15 ul

## 6 Efficiency test

Feed formulation prepared which includes: maize, wheat meal, soya bean cake and palm kernel cake, yeast, were feed into the mixer through the hopper, to obtain a homogenous mixture. The summation of the individual weight of ingredients gave a total weight of 6kg. After the mixing process, the homogenous mixture obtained was weighed to be 4.5kg.The efficiency of the feed mixer is thus calculated as:

Totalweightfeedin=6kg

weightofhomogenousmixturerecovered=4.5kg

$$Efficiency\;of\;the\;mixer\;=\;\frac{4.5}{6}\times 100\;=\;75\%$$

$$Mechanical\;damage=\;\left(\frac{1.5}{6}\right)\times 100\;=\;25\%$$

With an efficiency and mechanical damage of 75 % and 25 % respectively, the machine stands to be a replacement to crude mixing methods hence, improving productivity.

### 6.1 Effect of process factors on homogeneity

The Central Composite Design (CCD) tool of the Design-Expert software was used to ascertain the effect of mix time and speed on the homogeneity of the fish feed obtained. The actual design matrix, showing the process factors and response obtained from the software is shown in Table 7.

**Table 7:** Design matrix for CCD.

Std	Factor 1 Speed (rpm)	Factor 2 Time (Mins)	Response Homogeneity (%)
1	100	5	87.78
2	300	5	88.9
3	100	25	93.89
4	300	25	94.1
5	100	15	91.81
6	300	15	91.43
7	200	5	91.19
8	200	25	96.15
9	200	15	96.79
10	200	15	96.32
11	200	15	95.85
12	200	15	96
13	200	15	95.72

The analysis of variance (ANOVA) table is presented in Table 8. The ANOVA gave a significant model F-value of 36.35 and a non-significant lack of fit F-value of 6.03. This indicates that the design matrix is capable of predicting the effect of the process parameters on the response. The final quadratic model is presented in Eq. (9). It comprises of the relevant terms which are time (B), the square of speed (A^2^), and the square of time (B^2^).

(9) $$Homogeneity=\;+73.25+0.76B-0.00035A^{2}-0.015B^{2}$$

**Table 8:** ANOVA for Response Surface Quadratic model.

Source	Sum of Squares	df	Mean Square	F-Value	p-value Prob > F	
Model	105.30	5	21.06	36.35	<0.0001	significant
A-Speed	0.15	1	0.15	0.26	0.6261	
B-Time	44.12	1	44.12	76.14	< 0.0001	
AB	0.21	1	0.21	0.36	0.5688	
A^2^	34.69	1	34.69	59.87	0.0001	
B^2^	6.16	1	6.16	10.64	0.0138	
Residual	4.06	7	0.58			
Lack of Fit	3.32	3	1.11	6.03	0.0577	not significant
Pure Error	0.73	4	0.18			
Cor Total	109.35	12				

**Figure 14:**
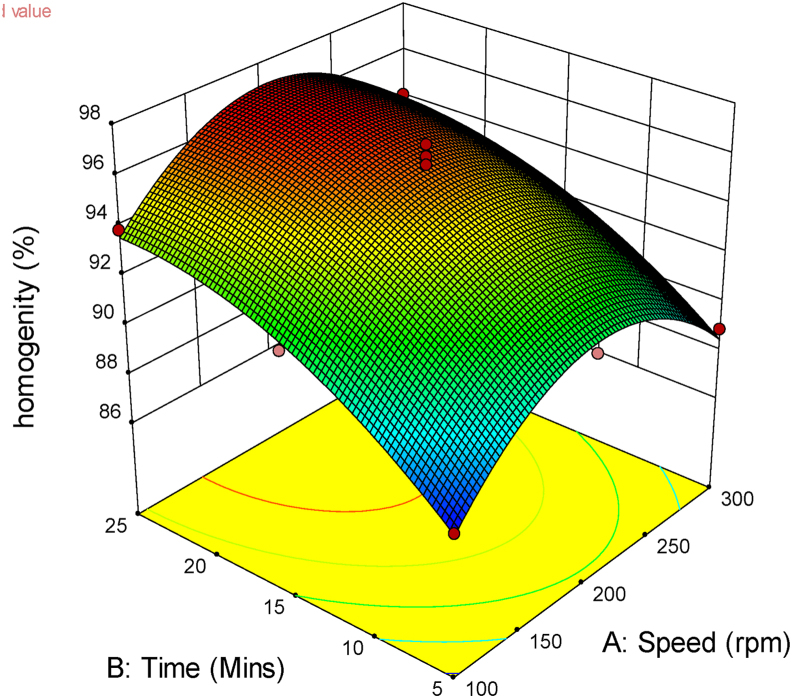
3D surface plot of time against speed for homogeneity.

**Figure 15:**
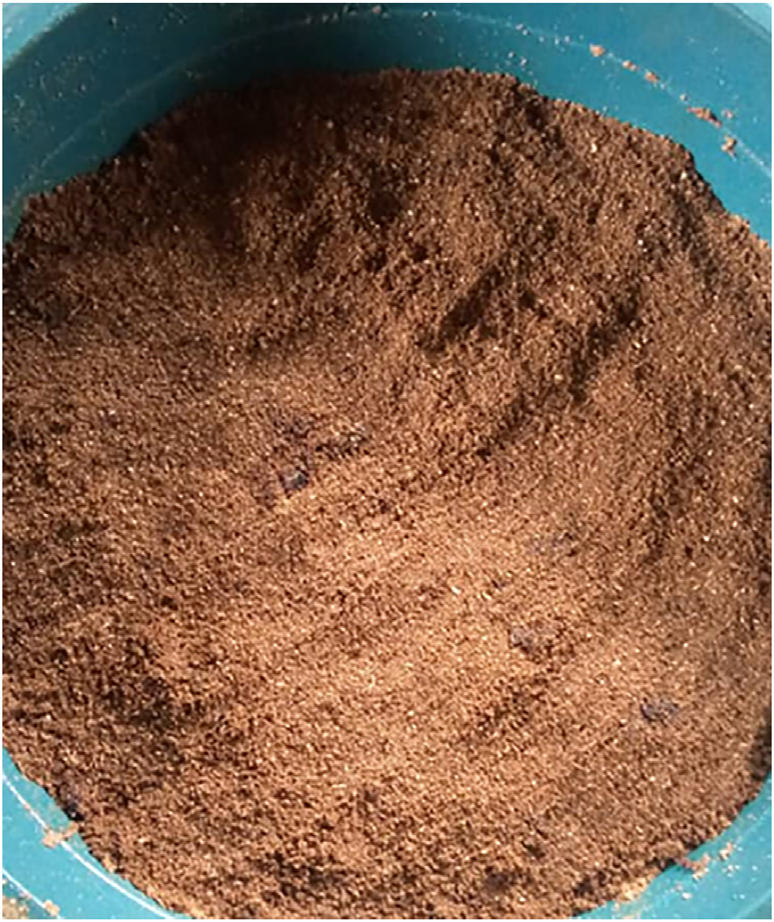
Fish feed after mixing.

The effect of the process parameters, mixing time, and speed is captured in Figure 14. It could be seen from the figure that mixing time has a positive effect on the homogeneity of the mixture (homogeneity increases with increasing mixing time). Following the plot, maximum homogeneity will be achieved with 25 minutes of mixing. Also, the plot reflects an increasing homogeneity up to a speed of 220 rpm beyond which retardation in the homogeneity of the feed will be recorded. These effects of mixing time and speed obtained from this study agree with the result of Adenigba and Olalusi (2019). The image of the obtained mixture is shown in Figure 15.

## 7 Conclusion

The fish feed mixer was produced by putting to play standard design procedures and effective material. The mixer runs with a 1.5Hp, single-phase electric motor and has a production capacity of 50 kg/h. Simulation of the mixer was conducted using a maximum test load of 6kg (equivalent force of 58.86N) of the mixture. The aforementioned force was evenly distributed along the length of the shaft and in 3 dimensions (i.e. x, y, and z directions). The simulation setup involves an imposed motion of 360 deg./sec, resistant frictional force coefficient of 0.5, simulation time of 4 s, and interval of 100. A maximum von mises stress of 53.7697 MPa, obtained which is much less than the yield strength of the conveyor assembly, indicates that the assembly will not fail.

Other results obtained include the imposed motion (driving torque), force, and moment, the highest values of the above results were exported individually to the finite element environment for thorough study. The FEA study shows that the design of the mixing shaft is suitable for the purpose it was designed for since the Von Mises results are well below the yield strength of the material. After a thorough mechanical test, an efficiency of 75 % was obtained for the machine. Hence, this device can be effectively introduced to fish feed production to increase productivity.

Through the integration of an electronic temperature regulator in the machine's control unit, an improvement of the quality of pellets produced was ensured. A major goal of making an environmentally friendly machine was achieved as the machine uses a clean power source and produces fish feed pellets of various sizes, shapes, and formulae depending on the choice of what to produce.

## Declarations

### Author contribution statement

Paul Chukwulozie Okolie, Echezona Nnaemeka Obika, Benjamin Segun Oluwadare, Onyemazuwa Andrew Azaka & Uchenna Onyebuchi Okolie: Conceived and designed the experiments; Performed the experiments; Analyzed and interpreted the data; Contributed reagents, materials, analysis tools or data; Wrote the paper.

### Funding statement

This research did not receive any specific grant from funding agencies in the public, commercial, or not-for-profit sectors.

### Data availability statement

Data included in article/supp. material/referenced in article

### Declaration of interests statement

The authors declare no conflict of interest.

### Additional information

No additional information is available for this paper.
